# Introduction of Inactivated Poliovirus Vaccine and Impact on Vaccine-Associated Paralytic Poliomyelitis — Beijing, China, 2014–2016

**DOI:** 10.15585/mmwr.mm6649a4

**Published:** 2017-12-15

**Authors:** Dan Zhao, Rui Ma, Tao Zhou, Fan Yang, Jin Wu, Hao Sun, Fang Liu, Li Lu, Xiaomei Li, Shuyan Zuo, Wei Yao, Jian Yin

**Affiliations:** ^1^Beijing CDC, China; ^2^Shijingshan District of Beijing CDC, China; ^3^Dongcheng District of Beijing CDC, China; ^4^Chaoyang District of Beijing CDC, China; ^5^World Health Organization Country Office, Beijing, China; ^6^Shenzhen Jin Wei Xin Technology Co., LTD, Shenzhen City, China.

When included in a sequential polio vaccination schedule, inactivated polio vaccine (IPV) reduces the risk for vaccine-associated paralytic poliomyelitis (VAPP), a rare adverse event associated with receipt of oral poliovirus vaccine (OPV). During January 2014, the World Health Organization (WHO) recommended introduction of at least 1 IPV dose into routine immunization schedules in OPV-using countries (*1*). The Polio Eradication and Endgame Strategic Plan 2013–2018 recommended completion of IPV introduction in 2015 and globally synchronized withdrawal of OPV type 2 in 2016 (*2*). Introduction of 1 dose of IPV into Beijing’s Expanded Program on Immunization (EPI) on December 5, 2014 represented China’s first province-wide IPV introduction. Coverage with the first dose of polio vaccine was maintained from 96.2% to 96.9%, similar to coverage with the first dose of diphtheria and tetanus toxoids and pertussis vaccine (DTP) (96.5%–97.2%); the polio vaccine dropout rate (the percentage of children who received the first dose of polio vaccine but failed to complete the series) was 1.0% in 2015 and 0.4% in 2016. The use of 3 doses of private-sector IPV per child decreased from 18.1% in 2014, to 17.4% in 2015, and to 14.8% in 2016. No cases of VAPP were identified during 2014–2016. Successful introduction of IPV into the public sector EPI program was attributed to comprehensive planning, preparation, implementation, robust surveillance for adverse events after immunization (AEFI), and monitoring of vaccination coverage. This evaluation provided information that helped contribute to the expansion of IPV use in China and in other OPV-using countries.

OPV has been employed in China’s EPI system for decades, leading to certification of China’s polio-free status in 2000.* After elimination of wild-type polio in China, VAPP, a rare occurrence of paralysis associated with a mutated vaccine virus that occurs in an OPV recipient or a close unvaccinated or nonimmune contact of the OPV recipient, emerged as an unacceptable risk: during 2010–2014, an average of one case of VAPP (reporting rate 7.16 per million first OPV doses) occurred annually among previously healthy children in Beijing. A majority of VAPP occurs in infancy, associated with the first OPV dose (*1*). IPV provides immunity against wild polioviruses, but cannot cause VAPP and greatly reduces the risk for VAPP associated with subsequent OPV doses. Countries that have previously introduced at least 1 IPV dose before vaccination with OPV have rapidly eliminated VAPP (*1*). IPV has been available in China's private sector since 2009. After completion of immunogenicity studies (*3*–*5*), Beijing introduced IPV into the public sector EPI program in December 2014 as part of a sequential schedule that included 1 dose of IPV at age 2 months, followed by 3 doses of trivalent OPV at ages 3, 4, and 48 months. After the global synchronized withdrawal of all Sabin type 2 vaccines in April 2016, trivalent OPV was replaced with bivalent OPV, which contains types 1 and 3 oral polio vaccine viruses.

Preparation for IPV introduction included addressing financial constraints, establishing a management structure, and developing an operational plan. The Beijing municipal government secured RMB18.9 million yuan ($US 2.9 million) for IPV procurement and program operations. During April–November 2014, the Beijing provincial health authorities developed a comprehensive work plan with technical guidelines for cold chain capacity assessment, training, risk communication, frequently asked questions, logistics materials (e.g., vaccines, forms), supply and distribution, and surveillance for polio vaccine utilization and AEFIs. During November 2014, health authorities issued an official circular that detailed responsibilities of various agencies and stated an objective to achieve 98.0% coverage with IPV. Information about the new IPV/OPV schedule was disseminated through the Beijing Municipal Authority’s website. Posters describing IPV and the availability of free vaccinations were posted on December 5, 2014, the first day that government-supplied IPV was offered. Health care workers were the primary sources of information about IPV introduction. Health care workers in vaccination clinic training workshops focused on immunogenicity, safety, and risk communication regarding the sequential schedule. Training materials included a polio fact sheet with frequently asked questions for parents, the new immunization schedule, eligibility criteria for IPV catch-up vaccination, and correct vaccine administration technique. Training was completed 2–7 days before IPV was introduced.

In December 2015, a program evaluation was conducted at the provincial level CDC (Beijing CDC), four subordinate district level CDCs, and 12 health facilities, by using the WHO Post Introduction Evaluation (PIE) tool (*6*). This tool is a systematic method for evaluating the effect of introducing a vaccine on a country’s existing immunization system. Beijing CDC surveyed 83 health care workers who were vaccinating children and 40 parents or guardians whose children were offered IPV. Polio vaccine utilization data were obtained from Beijing’s Immunization Planning Information System. Beijing CDC compared the proportions of eligible children receiving IPV and OPV before routine IPV introduction (December 2013–November 2014) and after IPV introduction (December 2014–November 2015) to assess utilization and preferences regarding polio vaccines and compared the polio vaccine and DTP dropout rates in 2015 and 2016 among children aged 1 year (born during October–November 2014 and 2015, respectively).

Adequate cold chain storage capacity was identified in all 12 surveyed sites. In addition to manual temperature recording, nine of the 12 surveyed health facilities were using a system that alerts vaccine mangers of temperature excursions. Oversight regarding IPV introduction was incorporated into routine supervision, with priority placed on vaccine usage and management. During the 6 months before the PIE, each surveyed health facility reported receiving 1–4 supervisory visits by district CDC personnel. Vaccine wastage data were reported by health facilities to district CDCs on a monthly basis. Median OPV and IPV wastage rates were 2.3% (range = 0%–5.3%) and 0.03% (range = 0%–1.2%), respectively.

Among the 83 health care worker survey respondents, 77 (93%) received training, and 80 (96%) responded correctly to questions about the immunization schedule, proper injection technique, contraindications to vaccination, and common AEFIs; all health care workers knew the appropriate anatomic site for injecting IPV. At least two of the following messages were relayed to parents by 72 (87%) health care workers: the vaccine name, the disease prevented, the sequential IPV/OPV schedule, the benefits of IPV, common AEFIs, how to report AEFIs, and the need to bring the child’s vaccination card to each visit. Among 40 parents or guardians whose children were offered IPV at the health facility, 13 (33%) knew what IPV and poliomyelitis were; among these 13 persons the primary sources of information about IPV were health care workers (seven), the Internet (four), and friends or relatives (two).

All surveyed sites reported that they had sufficient IPV and ancillary supplies (e.g., registration forms, certificates). Although new vaccination cards that included the IPV/OPV sequential schedule were issued to replace the previous cards, five (12.5%) surveyed parents still had the older vaccination cards on which IPV doses were recorded. Used needles and syringes were observed to have been discarded into safety boxes without recapping. Also, in five of the 12 health facilities, health care workers were observed to frequently manually disconnect the needle from syringe.

The existing acute flaccid paralysis (AFP) surveillance system, which needs to be sensitive enough to detect one case of AFP per 100,000 children aged <15 years, even in the absence of polio, has detected from 1.1 to 2.3 nonpolio AFP cases per 100,000 children aged <15 years annually during 2010–2016 in Beijing. VAPP cases were initially detected through this system. Since IPV introduction, clinicians, IPV suppliers, and district CDCs have reported any AEFI, including VAPP, after IPV administration through the existing passive, online AEFI surveillance system (*7*). During the first 2 years after IPV introduction, 115 mild adverse events (fever, local reaction, rash, or angioneurotic edema) and two rare adverse reactions (one case each of anaphylactoid purpura and thrombocytopenic purpura [both patients fully recovered]) were recorded. In addition, 22 adverse events that were determined, after expert panel review, to be unrelated to vaccination (i.e., coincidental events) occurred. These coincidental events included infections, allergies, thrombocytopenia, and infantile spasms. No case of VAPP has been reported since 2014 (Figure 1).

**FIGURE 1:**
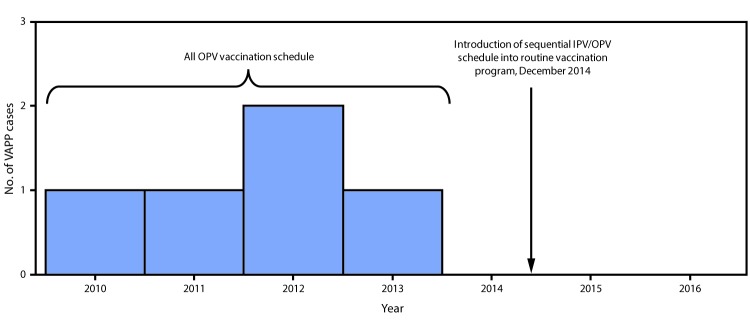
Vaccine-associated paralytic poliomyelitis (VAPP) cases identified through acute flaccid paralysis surveillance, by year — Beijing, 2010–2016 **Abbreviations:** IPV = inactivated polio vaccine; OPV = oral poliovirus vaccine.

Administrative coverage rates with the first dose of polio vaccines during 2014, 2015, and 2016 were 96.2%, 96.9%, and 97.4%, respectively; these rates were similar to those for the first DTP dose during those years (96.5% [2014], 97.2% [2015], and 97.6% [2016]). The polio vaccine drop-out rate was 1.0% in 2015 and 0.4% in 2016, similar to that for DTP (1.5% [2015], 2.1% [2016]). Before introduction of the sequential IPV/OPV schedule in Beijing, parents could choose IPV or an IPV-containing combination vaccine, such as Pentavalent *(*Pentaxim, Sanofi Pasteur, France) (which protects against diphtheria, tetanus, pertussis, polio, and *Haemophilus influenzae* type b) for the second or third polio vaccine dose, at their expense. However, in June 2016, China’s national drug and health authorities prohibited IPV for all 3 doses in the private market because of a global IPV shortage, and to ensure that all children could get a first IPV dose.^†^ The use of 3 doses of private-sector IPV declined slightly from 18.1% in 2014 to 17.4% in 2015 and to 14.8% in 2016 (Figure 2).

**FIGURE 2:**
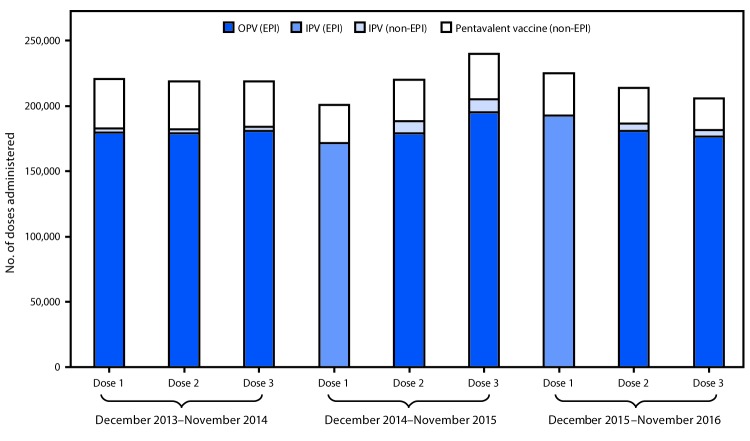
Poliovirus vaccine doses administered before* and after**^†^** the December 2014 introduction of inactivated polio vaccine (IPV) into the routine immunization program^§^ — Beijing, December 2013–November 2016 **Abbreviations:** EPI = expanded program on immunization; OPV= oral poliovirus vaccine. * December 2013–November 2014. ^†^ December 2014–November 2016. ^§^ The Beijing immunization program provide IPV and OPV in the sequential schedule without charge; however, parents can choose IPV for the second and third doses, or an IPV-containing combination vaccine at their own expense. IPV is standalone Salk-poliovirus strains; Pentavalent vaccine is a combination vaccine containing diphtheria, tetanus, acellular pertussis vaccine, inactivated poliovirus, and *Haemophilus influenzae* type b vaccines.

## Discussion

IPV introduction using a sequential IPV/OPV schedule in Beijing was associated with a good safety record, no occurrence of VAPP or other serious adverse events, and maintenance of >95% coverage with the first dose of polio vaccine. There was little change in the relatively small percentage of children receiving an all-IPV schedule through the private sector. Given the current global shortage of IPV (*8*), it was reassuring that public confidence in the safety of OPV remained high, assuring the availability of 1-dose IPV access. OPV wastage exceeded that of IPV, possibly because infants occasionally spat out the oral dose, which had to be repeated.

Strong public health leadership, good operational planning, secured resources, and budget were critical to successful IPV introduction in Beijing. However, the PIE did identify areas for improvement. For example, two thirds of parents interviewed were not familiar with either IPV or poliomyelitis, possibly because of the short time available for health workers to educate parents and still administer all vaccines. Because health care workers served as the primary sources of information about the sequential schedule to parents, there was a risk that the occurrence of any serious AEFIs might cause parents to lose confidence in the vaccination program, especially if a serious AEFI were to be widely reported by the media (*9*,*10*). Thus, large-scale media campaigns, describing the program, and monitoring public concerning the safety of polios vaccines should be reinforced during IPV introduction. In addition, compliance with safe injection practices by health care workers needs improvement through more targeted training. 

The findings in this report are subject to at least two limitations. First, as recommended in the PIE tool, 40 parents and 12 health facilities were selected for the survey; however, because of the large population in Beijing and the large annual birth cohort, the small sample might not be representative. Second, although no VAPP cases were reported during the 2 years after IPV introduction, additional time will be needed to assess the impact of the IPV/OPV sequential schedule on VAPP in Beijing.

Successful implementation of the sequential IPV/OPV schedule in Beijing and the findings of the PIE demonstrate the feasibility of implementing the sequential schedule throughout the country, and of introducing another injectable vaccine into the childhood immunization schedule. In addition, surveillance data regarding VAPP from the first 2 years after IPV introduction indicate that, as has been observed in other countries, if IPV is made available in a sequential schedule throughout China, VAPP could be eliminated.

**Summary**

**What is already known about this topic?**

Since 2014, the World Health Organization has recommended that all countries using oral poliovirus vaccine (OPV) introduce at least 1 dose of inactivated polio vaccine (IPV) into routine immunization programs. However, the evaluation of IPV introduction after this global recommendation was limited, including the impact that IPV introduction might have on the existing immunization program. Beijing Municipal Authority implemented the first province-wide IPV introduction in China on December 5, 2014 with a sequential IPV/OPV poliovirus vaccination schedule.

**What is added by this report?**

Two years after introduction of the sequential IPV/OPV vaccination schedule in Beijing, a postintroduction evaluation was conducted. The sequential schedule was successfully introduced into the public-sector Expanded Program on Immunization system and was well accepted by parents and providers. Compared with the year preceding IPV introduction, polio vaccination coverage remained high, no adverse effect on coverage with other vaccines occurred, and no cases of vaccine-associated paralytic poliomyelitis have been identified.

**What are the implications for public health practice?**

Comprehensive IPV introduction plans not only ensure a smooth transition to a new vaccine schedule, but can also help improve the current routine immunization system. Good planning and preparation can lead to high coverage with a new vaccine without negative impact on coverage with other vaccines. The experience in Beijing helped contribute to expansion of IPV use nationwide in China, and can also aid IPV introductions in other OPV-using countries.

## Footnotes

* http://www.wpro.who.int/china/mediacentre/factsheets/polio/en/.

† http://app1.sfda.gov.cn/WS01/CL1368/155900.html.
